# Different acupuncture therapies combined with rehabilitation in the treatment of scapulohumeral periarthritis: A protocol for systematic review and network meta-analysis

**DOI:** 10.1097/MD.0000000000023085

**Published:** 2020-12-18

**Authors:** Lingzhi Wei, Manhua Zhu, Tianzhong Peng, Wei Xiong, Xinju Hou

**Affiliations:** Nanchang Hongdu Hospital of Traditional Chinese Medicine, Jiangxi Province, China.

**Keywords:** acupuncture, network meta-analysis, protocol, rehabilitation, scapulohumeral periarthritis

## Abstract

**Background:**

Scapulohumeral periarthritis is a disease that seriously affects human daily work and life, and greatly reduces peoples quality of life and affects human health all over the world. Now, many studies have shown that acupuncture and rehabilitation have a significant effect on scapulohumeral periarthritis. In this study, network meta-analysis was used to analyze and compare the clinical efficacy and difference of different acupuncture treatments on scapulohumeral periarthritis.

**Methods:**

All patients were diagnosed as scapulohumeral periarthritis by randomized controlled trial. Computer searches will be conducted on CNKI, Wan-Fang databases, VIP, CBM, Pubmed, Cochrane library, Embase, Web of Science. The retrieval period is from the date of database establishment to September 8, 2020. To avoid omissions, we will manually retrieve relevant references and conference papers. Finally, the risk of bias included in the study will be assessed according to the guidelines of the Cochrane Handbook for systematic review of interventions. All data analysis will be performed by Revman 5.3, WinBUGS1.4.3 and Stata14.2.

**Results:**

The effectiveness of each intervention was quantified. The main results included cure rate, total effective rate, VAS score and shoulder function score.

**Conclusion:**

Objective to provide evidence-based medicine basis for clinicians to choose more effective acupuncture therapy for scapulohumeral periarthritis.

**INPLASY Registration number:**

202090035.

## 1 Introduction

Scapulohumeral periarthritis is a kind of aseptic inflammatory disease caused by degenerative changes and long-term strain of shoulder joint and its surrounding soft tissue.^[1]^ The disease is most common in manual workers aged 50 or above, and the incidence rate of female is slightly higher than that of men.^[2]^ The incidence rate is increasing, and it is one of the most common refractory diseases. Its clinical manifestations are mainly pain around the shoulder joint, dysfunction of joint function, chills, muscle spasm, and severe muscle atrophy.^[3]^ It can be said to a large extent affected the quality of life and daily work of patients and can even cause some psychological problems. At present, the commonly used treatment methods of scapulohumeral periarthritis include oral nonsteroidal analgesics, block therapy, surgery, etc.^[4]^ However, there are also some problems such as drug toxicity and surgical trauma and risk. So it is very important to find a more safe and effective treatment. Modern research shows that acupuncture and rehabilitation therapy can relieve pain, regulate blood circulation, enhance immunity and improve joint range of motion, so it is widely used in clinic.^[5–7]^

Systematic evaluation is an important evidence to guide clinical decision-making. More and more systematic studies have shown that acupuncture plays an important role in the treatment of scapulohumeral periarthritis.^[8–10]^ Now there are many kinds of acupuncture therapy, but the standard system evaluation function is limited, so it can only directly compare the intervention, and can not compare a variety of acupuncture therapy. The advantages and curative effects of various acupuncture therapies are not the same, so it is difficult for clinicians to choose which kind of acupuncture therapy and which kind of curative effect is better. Therefore, this study will rank the clinical efficacy of commonly used acupuncture and moxibustion therapy through network meta-analysis, so as to provide scientific evidence-based medicine for clinical optimization of the best acupuncture therapy.

## 2 Methods

### 2.1 Study registration

This systematic review protocol will be reported strictly adherence to the Preferred Reporting Items for Systematic Review and Meta-analysis Protocols (PRISMA-P).^[11]^ All study protocols must be funded through a protocol registry. This study protocol has been registered and approved on the INPLASY website. The registration number is INPLASY202090035, and it could be found at https://inplasy.com/inplasy-2020-9-0035/. Any changes in the full text will be described.

### 2.2 Inclusion criteria

#### 2.2.1 Study type

Based on different acupuncture and rehabilitation treatment of scapulohumeral periarthritis RCTs, the language is limited in Chinese and English. Literature exclusion criteria:

1. Non RCTs literature, such as case report, literature review, etc;
2. Besides 4 kinds of acupuncture, other acupuncture therapies were used;
3. The experimental group and the control group contained other interference therapy;
4. Only 1 document with the most complete information was selected for the repeated detection and repeated publication;
5. Literature with incomplete data or unable to obtain data and full text;
6. Documents suspected of counterfeiting.

#### 2.2.2 Participants

The patients diagnosed as scapulohumeral periarthritis meet the internationally recognized diagnostic criteria and have clear curative effect criteria. There are no restrictions on age, race, gender, and source of cases. However, the following patients will be excluded:

1. Patients who can not tolerate acupuncture and moxibustion treatment;
2. Patients with severe organic diseases;
3. Pregnant women;
4. Patients with mental illness or inability to accurately describe symptoms due to unconsciousness.

#### 2.2.3 Interventions

The experimental group was treated with electroacupuncture, fire acupuncture, warm acupuncture, or floating acupuncture. The control group was treated with simple rehabilitation therapy. Both the experimental group and the control group could cooperate with conventional medical treatment.

#### 2.2.4 Outcome indicators

The included outcome indicators included 1 or more of the following: cure rate, total effective rate (effective rate = ([Cured + markedly effective + effective]/total number of cases ×100%), visual analogue pain scale (VAS) score, shoulder joint function score.^[12,13]^

### 2.3 Data sources and search strategies

**Figure 1:**
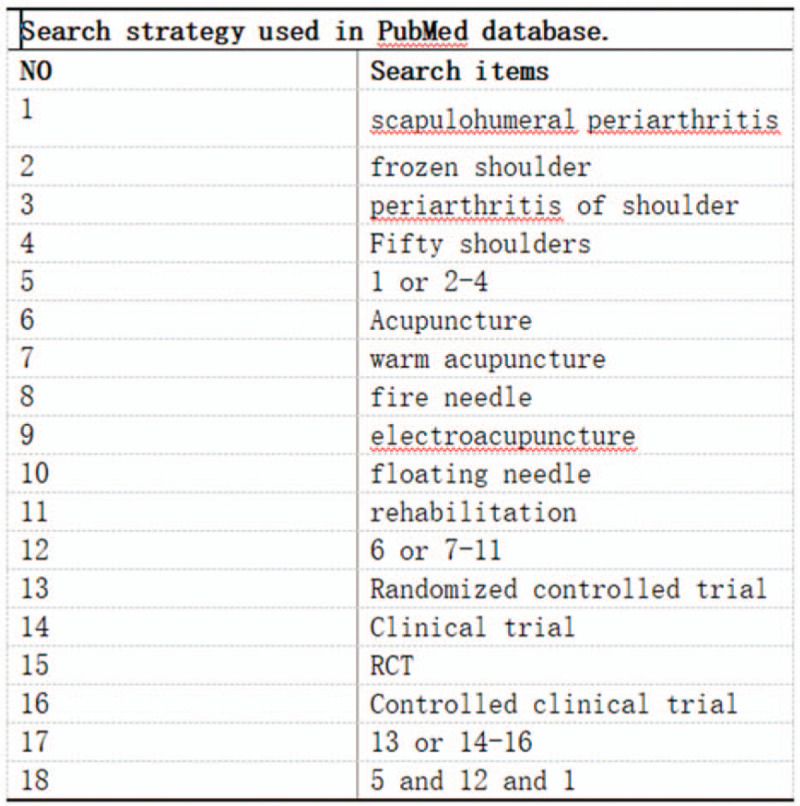
Search strategy used in PubMed database.

Chinese databases including CNKI, Wan-Fang databases, VIP and SinoMed were searched by computer. PubMed and Cochrane library, Embase, Web of Science were searched in English database. The key words are “warm acupuncture”, “fire needle”, “electroacupuncture”, “floating needle”, “periarthritis of shoulder”, “frozen shoulder”, “periarthritis of shoulder”, and “Fifty shoulders”. The retrieval time is from the establishment of the database to September 8, 2020. All of the retrieval strategies are in the form of subject words + free words, and relevant references are retrieved manually. The retrieval strategy is shown in Figure 1.

### 2.4 Selection of studies and data extraction

Two reviewers screened independently according to the inclusion and exclusion criteria, and then cross checked. In case of disagreement, it shall be decided by a third evaluator. The information extraction table was established in Excel. The extracted information included: author, publication time, number of cases, allocation method, intervention measures, course of treatment and outcome indicators.

### 2.5 Risk assessment of bias

Two reviewers independently assessed the bias risk of the articles included in this study according to the Cochrane evaluator bias risk assessment tool. It includes selection bias, implementation bias, measurement bias, follow-up bias, reporting bias and other sources bias. The evaluation results were evaluated as “high risk”, “low risk”, and “unclear risk”.^[14]^

### 2.6 Statistical analysis

We used Revman 5.3 software for bias evaluation and direct meta-analysis. The outcome indicators were the ratio ratio (or) of count data, the mean difference (MD) of measurement data, and the 95% confidence interval (95% CI) for the effect.

In the heterogeneity test, if *I*^2^ < 50%, *P* > .10, there is no significant heterogeneity. We chose the fixed effect model to combine the effect quantity. If the combined data is *I*^2^ > 50%, *P* < .10, it indicates high heterogeneity. We choose the random effect model to combine the effect quantity.^[15]^

We used WinBUGS1.4.3 and Stata14.2 for mesh meta-analysis.^[16,17]^ In WinBUGS software, the method of Markov chain Monte Carlo (MCMC) is used for Bayesian network meta-analysis. It is simulated through 4 chains, and the number of iterations is set to 50,000, the first 20,000 annealing times are used to eliminate the influence of initial value and the step size is set to 10.^[18]^ At the same time, it uses potential scale reduced factor (PSRF) to evaluate the convergence of the results. When the PRSF is close to or equal to 1.00 (1.00 ≤ PRSF ≤ 1,05), the results show good convergence and high reliability.^[19]^

### 2.7 Assessment of inconsistency

There are many interventions involved in this study. In the evidence network of each outcome index, the closed loop formed by the research with direct evidence and indirect evidence needs to be assessmented of inconsistency by Stata software. The inconsistency factor (IF) was calculated, and the inconsistency was judged by the size of IF value and *P* value.^[20]^ If the IF is close to 0, 95% CI starts at 0, and *P* > .05, the results of direct comparison and indirect comparison are consistent. At the same time, it uses the node split model to determine whether each node has local inconsistency.^[21]^ If *P* > .05, consistency model is used; otherwise, inconsistency model is used.^[22,23]^

In Stata software, calculate the SUCRA (surfaceunder the cumulative ranking curves, SUCRA) value and area under the curve by sucra prob command.^[24]^ It is convenient to sort the effects of various interventions. It ranges from 0 to 100. The larger the value and the larger the area under the curve, the better the effect of the intervention measures.

### 2.8 Sensitivity analysis

The purpose of sensitivity analysis is to eliminate low-quality research and explore the sources of heterogeneity.^[25]^ Then, the reliability and stability of the results were analyzed by observing the heterogeneity of different studies and whether the results changed after treatment.

### 2.9 Assessment of publication bias

If outcome indicators are included in the study ≥10, funnel plot will be used to assess publication bias for inclusion in the trial.^[26]^ If there are differences in symmetry or distribution, there will be publication bias or small sample effect.

## 3 Ethics and dissemination

Because this is a systematic review of the protocol and a network meta-analysis, all the data in this study are from published studies and do not involve patients, so there is no need for ethical recognition. The results of this study will be distributed to peer reviews and presented at relevant meetings.

## 4 Conclusion

Now, many systematic reviews of acupuncture therapy for scapulohumeral periarthritis show that acupuncture is effective in treating scapulohumeral periarthritis. But there are many kinds of acupuncture in clinic, and the treatment advantages are different. Therefore, whether acupuncture can be used as the dominant therapy for scapulohumeral periarthritis and which acupuncture has better curative effect is lack of accurate evidence-based evidence. However, network meta-analysis can overcome the shortcomings of standard meta-analysis and integrate direct and indirect evidence, and it can provide an intuitive comparison of the effectiveness and safety of existing technologies. Therefore, we will use the network meta-analysis method to evaluate the curative effect of 4 commonly used acupuncture techniques, and the curative effect was quantified according to different therapeutic indexes. These results can provide the basis for clinicians to determine the treatment plan for patients with scapulohumeral periarthritis.

## Author contributions

CQ, XT, YM, XX, MW, YD, JZ and PL analyzed the case and YY designed the study. All authors participated in writing and approved the manuscript.

**Conceptualization:** Lingzhi Wei, Xinju Hou.

**Data curation:** Tianzhong Peng.

**Formal analysis:** Manhua Zhu.

**Project administration:** Lingzhi Wei, Wei Xiong, Xinju Hou.

**Supervision:** Lingzhi Wei, Xinju Hou.

**Writing – original draft:** Lingzhi Wei.

**Writing – review & editing:** Lingzhi Wei, Manhua Zhu, Xinju Hou.

## References

[1] Prescher A Anatomical basics, variations, and degenerative changes of the shoulder joint and shoulder girdle. Eur J Radiol 2000;35:88–102. 10963915 10.1016/s0720-048x(00)00225-4

[2] Mubark IM Ragab AH Nagi AA Evaluation of the results of management of frozen shoulder using the arthroscopic capsular release. Ortop Traumatol Rehabil 2015;17:21–8. 25759152 10.5604/15093492.1143530

[3] Fan X Cheng B Huang Y Immediate effects of Hegu needling on adhesive scapulohumeral periarthritis. J Acupunct Tuina Sci 2013;11:258–61.

[4] Kraal T Visser C Sierevelt I How to treat a frozen shoulder? A survey among shoulder specialists in the Netherlands and Belgium. Acta Orthop Belg 2016;82:78–84. 26984658

[5] Jie W Fang X Zhang A Buccal acupuncture plus exercise therapy for scapulohumeral periarthritis. J Acupunct Tuina Sci 2016;14:131–4.

[6] Zhao Z Jiang G Chen S Treatment of 46 cases with scapulohumeral periarthritis by acupuncture plus Shi's Twelve-word Life Cultivation Skill. J Acupunct Tuina Sci 2012;10:191–5.

[7] Oleinikov BV Knyazhishche AN Oleinikov DB Myofascial meridional reflexotherapy of scapulohumeral periarthrosis in the course of the combined spa and health resort-based treatment. Vopr Kurortol Fizioter Lech Fiz Kult 2015;92:45–7. 10.17116/kurort2015645-47 26841529

[8] Shi H Fang JQ Li BW Efficacy assessment for different acupuncture therapies in the treatment of frozen shoulder. World J Acupunct 2012;22:6–11.

[9] Ji L Wang H Cao Y Sharp-Hook acupuncture (Feng Gou Zhen) for patients with periarthritis of shoulder: a randomized controlled trial. Evid-Based Compl Alt 2015;2015:312309. 10.1155/2015/312309 PMC4657093 26640496

[10] Yuan Q Wang P Liu L Acupuncture for musculoskeletal pain: a meta-analysis and meta-regression of sham-controlled randomized clinical trials. Sci Rep-Uk 2016;6:30675. 10.1038/srep30675 PMC4965798 27471137

[11] Moher D Shamseer L Clarke M Preferred reporting items for systematic review and meta-analysis protocols (PRISMA-P) 2015 statement. Syst Rev 2015;4:1. 25554246 10.1186/2046-4053-4-1 PMC4320440

[12] Mei J Xiaoxia W Meijuan C The efficacy of acupuncture for the treatment of sciatica: a systematic review and meta-analysis. Evid Based Complement Alternat Med 2015;59:29–30. 10.1155/2015/192808 PMC4575738 26425130

[13] Kirkley A Griffin S Dainty K Scoring systems for the functional assessment of the shoulder. Arthroscopy 2003;19:1109–20. 14673454 10.1016/j.arthro.2003.10.030

[14] Savović J Weeks L Sterne JA Evaluation of the Cochrane Collaboration's tool for assessing the risk of bias in randomized trials: focus groups, online survey, proposed recommendations and their implementation. Syst Rev 2014;3:37. 24731537 10.1186/2046-4053-3-37 PMC4022341

[15] Huedomedina TB Sanchezmeca J Marinmartinez F Assessing heterogeneity in meta-analysis: Q statistic or I2 index? Psychol Methods 2006;11:193–206. 16784338 10.1037/1082-989X.11.2.193

[16] Stephenson M Fleetwood K Yellowlees A Alternatives to winbugs for network meta–analysis. Value Health 2015;18:A720.

[17] Chaimani A Higgins JP Mavridis D Graphical tools for network meta-analysis in STATA. PloS One 2013;8:e76654. 24098547 10.1371/journal.pone.0076654 PMC3789683

[18] Ades AE Sculpher M Sutton A Bayesian methods for evidence synthesis in cost-effectiveness analysis. Pharmacoeconomics 2006;24:1–9. 10.2165/00019053-200624010-00001 16445299

[19] Luccheta RC Riveros BS Pontarola R Systematic review and meta-analysis of the efficacy and safety of amfepramone and mazindol as a monotherapy for the treatment of obese or overweight patients. Clinics 2017;72:317–24. 28591345 10.6061/clinics/2017(05)10 PMC5439101

[20] Salanti G Ades AE Ioannidis JPA Graphical methods and numerical summaries for presenting results from multiple-treatment meta-analysis: an overview and tutorial. J Clin Epidemiol 2011;64:163–71. 20688472 10.1016/j.jclinepi.2010.03.016

[21] Dias S Welton NJ Caldwell DM Checking consistency in mixed treatment comparison meta-analysis. Stat Med 2010;29:932–44. 20213715 10.1002/sim.3767

[22] Song F Clark A Bachmann MO Simulation evaluation of statistical properties of methods for indirect and mixed treatment comparisons. Bmc Med Res Methodol 2012;12:138. 22970794 10.1186/1471-2288-12-138 PMC3524036

[23] Sturtz S Bender R Unsolved issues of mixed treatment comparison meta-analysis: network size and inconsistency. Res Synth Methods 2012;3:300–11. 26053423 10.1002/jrsm.1057

[24] Rücker G Schwarzer G Ranking treatments in frequentist network meta-analysis works without resampling methods. Bmc Med Res Methodol 2015;15:58. 26227148 10.1186/s12874-015-0060-8 PMC4521472

[25] Copas J Shi JQ Meta-analysis, funnel plots and sensitivity analysis. Biostatistics 2000;1:247–62. 12933507 10.1093/biostatistics/1.3.247

[26] Sutton AJ Duval SJ Tweedie RL Empirical assessment of effect of publication bias on meta-analyses. BMJ 2000;320:1574–7. 10845965 10.1136/bmj.320.7249.1574 PMC27401

